# Scoping Review: Evaluation of *Moringa oleifera* (Lam.) for Potential Wound Healing in In Vivo Studies

**DOI:** 10.3390/molecules27175541

**Published:** 2022-08-28

**Authors:** Nurmaziah Mohammad Shafie, Raja Nazatul Izni Raja Shahriman Shah, Puspawathy Krishnan, Noorashikin Abdul Haleem, Terence Yew Chin Tan

**Affiliations:** Herbal Medicine Research Centre, Institute for Medical Research, National Institutes of Health, Ministry of Health Malaysia, Persiaran Setia Murni U13/52, Bandar Setia Alam, Shah Alam 40170, Selangor, Malaysia

**Keywords:** *Moringa oleifera*, wound healing, merunggai, epithelialization, herbal medicine

## Abstract

Wound healing is a natural process to restore damaged tissues due to loss of tissue integrity. *Moringa oleifera* (locally known as merunggai in Malaysia) has been traditionally used in various ailments, including for wound management. To evaluate the wound healing properties in *M. oleifera*, publications were searched and selected following the guidelines of the Preferred Reporting Items for Systematic Reviews and Meta-Analyses (PRISMA) statement with predetermined inclusion criteria. The databases searched for primary studies include PubMed, Google Scholar, Science Direct, LILACS, ClinicalTrials.gov, and CENTRAL. In total, 18 in vivo studies were included, which involved the leaves, while the remaining 5 studies involved other plant parts tested on excision, incision, dead space, abrasion, and burn-induced wound models. All studies reported significant wound healing abilities. Most studies used different topical formulations of aqueous leaves extract. The accumulation of collagen content and underlying wound healing mechanism through antimicrobial, antioxidant, and anti-inflammatory activities may be contributed by its bioactive phytochemical content, which has the potential to accelerate the wound contraction, increase the rate of epithelialization, and protect tissues against oxidative damage. In conclusion, *M. oleifera* showed wound healing potential but further studies are warranted to determine the main bioactive phytocompounds and safety.

## 1. Introduction

Wound healing is a natural process whereby damaged tissues are repaired. It happens in four overlapping stages, namely haemostasis (blood clotting), inflammation, proliferation, and tissue remodeling [1,2,3]. This involves complex processes carried out by different types of cells, such as keratinocytes, fibroblasts, inflammatory cells, and endothelial cells [4]. Over the centuries, humans from all over the world have used various methods to treat wounds and the advent of modern wound healing only started in the 20th century [5].

It is estimated that wound care annual costs with an average of USD 2.8 billion in 2014 will increase to USD 3.5 billion by 2021 [6]. There are many factors impacting wound healing, such as oxygenation, infection, age and sex hormones, stress, diabetes, obesity, medications, alcoholism, smoking, and nutrition [2]. A five-year mortality study on patients with diabetic foot ulcers showed a comparable mortality rate with cancer (30.5% vs. 31%), indicating the impact of wounds to healthcare [7]. In order to reduce the wound burden towards healthcare and the economy, new therapeutic approaches and technologies are continually being developed [8]. At the same time, studies to evaluate the efficacy of documented traditional approaches for wound healing, such as herbs [9,10,11,12,13], or other alternative methods, such as leech therapy [14,15], are also conducted. An ideal wound dressing should be non-toxic and cost effective. Herbal medicine can be considered to have a non-toxic nature due to its long history of use and affordability [16,17]. *Moringa oleifera* is among the medicinal plants documented to be traditionally used for wound healing purposes.

*M. oleifera* is locally known as merunggai (Malay) in Malaysia or drumstick tree or radish tree elsewhere. *M. oleifera* is a member of the Moringaceae family. It is a well-known plant in Malaysia, India, Pakistan, Bangladesh, and Afghanistan, which was utilized for various purposes by ancient Romans, Greeks, Egyptians, and many tropical and subtropical countries, even until today [18,19,20]. *M. oleifera* is now spread throughout the tropics and is mostly found wild in Northern India [21]. The plant can grow up to 3–10 m tall and the bark has a grainy fiber inside and corky outside. The leaves are green, 2–3 pinnate in shape, and about 60 cm long. The flowers are fragrant and white in colour. The fruits are long and oval in shape, green when young, and turn to brown when older [18].

Traditionally, leaf paste of *M. oleifera* is used for wound healing in India [22]. In Malaysia, the plant’s root has been used variably for women’s health during confinement periods and the seed oil is applied onto the joints to treat rheumatism [23]. Other reported traditional uses include applications as a poultice on the abdomen to expel intestinal worms, rubbing over the breasts to prevent milk flow, orally consumed to treat gonorrhoea, and treating dropsy by mixing the leaves with lime [21,23]. The phytochemical content of *M. oleifera* leaves consists of polyphenol, phenolic acids, vitamins, carotenoids, isothiocyanates, tannins, saponins, flavonoids, alkaloids, glucosinolates, oxalates, and phytates, which are beneficial bioactive compounds [24]. Efficacy studies of *M. oleifera* have shown the plant as an antiseptic, antimicrobial, antispasmodic, antiulcer, antitumor, antihyperthyroidism, antihypertensive, and hepatoprotective agent [25,26,27,28,29].

Based on the traditional uses of *M. oleifera* in wound healing and its pharmacological action, such as antiseptic and antimicrobial, the objective of this study is to evaluate and present the scientific evidence pertaining to the wound healing potential of *M. oleifera* in different types of wounds. 

## 2. Results

### 2.1. Study Inclusion

**Figure 1:**
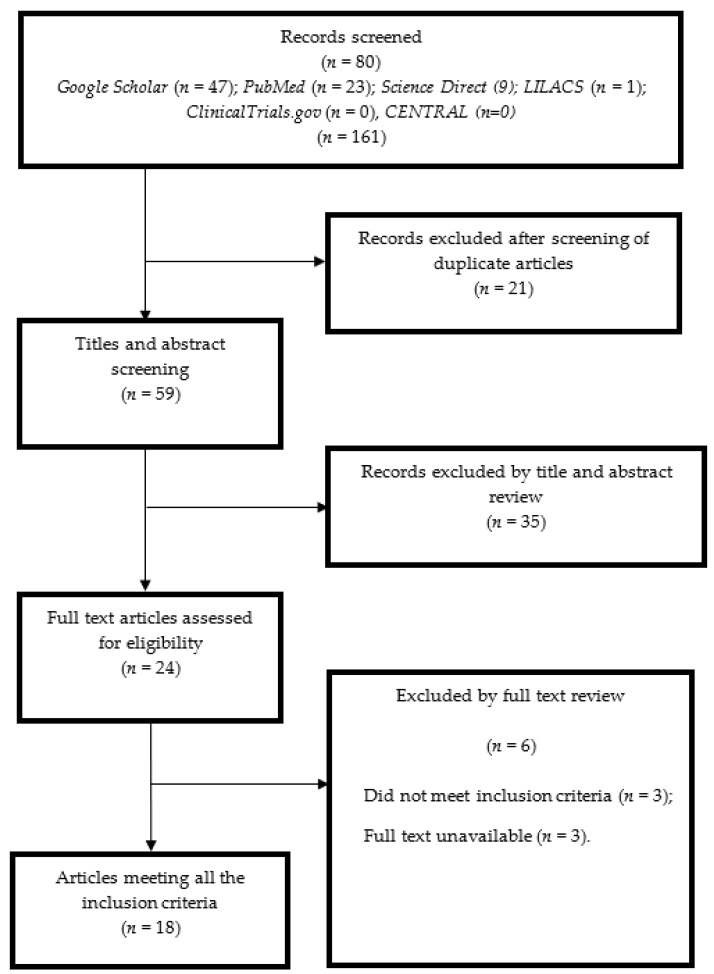
Preferred Reporting Items for Systematic reviews and Meta-Analyses.

Our search from the six databases produced a total of 80 related articles. After removing duplicates, 59 articles were screened and 18 articles were included [30,31,32,33,34,35,36,37,38,39,40,41,42,43,44,45,46,47], as summarized in Figure 1.

### 2.2. Characteristics of Included Studies

In total, 18 animal studies were included, which include six types of wounds model, i.e., excision, incision, dead space, abrasion, palatal, and burns. Eighteen included studies (Table 1) were examined. Nine out of the eighteen included studies were conducted in India (50%), three in Malaysia (17%), two in Indonesia (11%), and one study in Nigeria (6%), Brazil (6%), Philippines (6%), and Bangladesh (6%) respectively. Among the plant parts used are leaves (*n* = 13), bark (*n* = 2), twig (*n* = 1), fruits (pulp and seed, *n* = 1), and seed (*n* = 1). More than half of the studies used aqueous extract (55.56%), while others used ethanol (16.67%), methanol (5.56%), n-hexane (5.56%), ethyl acetate and ethanol (5.56%), aqueous and ethanol (5.56%) extracts. One study did not mention the type of extract used. Duration of treatment ranges from 10 days to 90 days. The study characteristics are presented in Table 1. 

### 2.3. Evidence of Wound Healing Activity

Majority of the studies indicate that *M. oleifera* was applied topically and formulated as gel (six studies [30,39,44,47] followed by using the extract in the form of paste, patch, or film dressing (five studies [30,33,36,40] and the remaining an ointment (three studies [34,41,42]. Two studies reported oral administration of *M. oleifera* aqueous extracts (two studies [38,43] while another two reported dual administration of both topical (ointment and aqueous extract) and oral (aqueous extracts) (two studies [32,37]. Among the included studies, 28% underwent an authentication process through voucher specimen deposition of the plant while another 39% indicated the plant was authenticated without a deposition reference number. None of the 18 studies reported qualitative analysis to determine the phytochemicals associated with *M. oleifera* or reported quantitative analysis to determine the composition of the associated phytochemicals in *M. oleifera.* Only two studies reported using a standardized formulation *M. oleifera* but details are incomplete [33,39]. Detailed information on the qualitative and quantitative phytochemical analysis, as well as standardization formula of the herbal interventions of all included studies, are presented in the Supplementary Material (Table S3). The data extraction of the intervention and findings of the in vivo studies of *M. oleifera* efficacy for wound healing are presented in Table 2.

### 2.4. Risk of Bias Assessment

**Figure 2:**
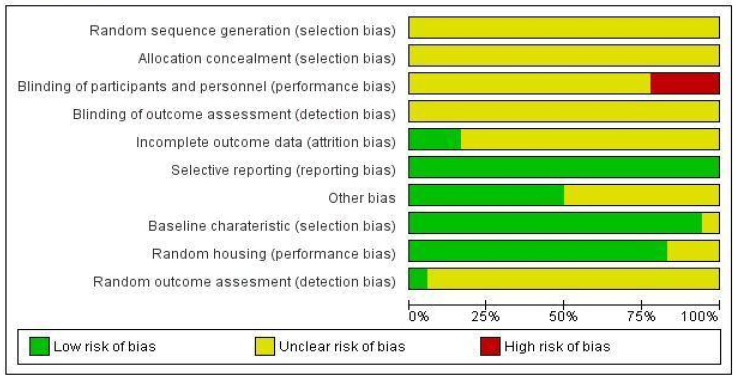
Risk of bias summary of included studies.

**Figure 3:**
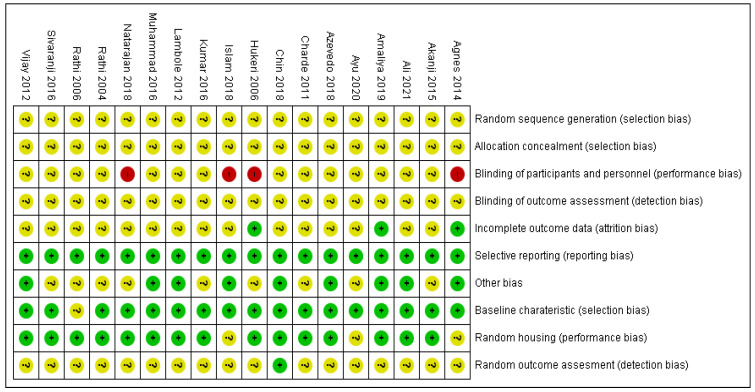
Risk of bias assessment of included studies of *M. oleifera* for wound healing: Green for low risk of bias, yellow for unclear risk of bias and red for high risk of bias.

Figure 2 and Figure 3 show the risk of bias assessment’s results for the 18 included studies. All studies have an unclear risk of bias on random sequence generation, allocation concealment, and blinding of outcome assessment, as all the studies did not report on these biases. More than 70% of the studies have unclear attrition bias (as incomplete outcome data) and detection bias (as random outcome assessment).

All studies (100%) showed a low risk of bias in selective reporting while more than 80% of the studies showed a low risk of bias for baseline characteristics and random housing of the animals. Further, 25% of the studies showed a high risk for performance due to non-blinding of the outcome assessment.

### 2.5. Safety Studies

During the data extraction of included studies, thirteen pieces of safety assessment data were extracted involving *M. oleifera* leaves. 

In terms of general toxicity, an acute oral toxicity study on an aqueous extract of *M. oleifera* leaves administered orally to male Swiss albino rats (18–22 g) found that LD_50_ was > 5000 mg/kg [38].

For specific toxicity, an acute dermal toxicity study observed on an *M. oleifera* leave-loaded hydrocolloid dressing administered to Sprague Dawley rats during 14 days of testing showed no mortality, no signs of oedema, erythema, or any symptoms of toxicity on animal skin. No abnormalities and no significant differences (*p* < 0.05) were detected on body-weight-gain percentage [33]. Skin irritation studies of aqueous extracts of *M. oleifera* leaves hydrogel (500 mg/animal) administered topically on excision wound of Wistar rats (200–250 g) twice a day for 7 days showed no skin irritation signs during the whole period of study [47]. A skin irritation test carried out using a mixture of aqueous extracts of *M. oleifera* leaves (MO) and human amniotic membrane (AM)-formulated gel (2% MO + AM) administered topically on wounds on female Wistar rats for 7 days did not show any skin oedema, itchiness, or erythema, suggesting tolerable dermal application [39]. A skin patch/scratch and skin sensitization test carried out using an ethanol extract of *M. oleifera* twigs (5 mg/mL, 7.5 mg/mL and 10 mg/mL) on a wound area of healthy guinea pigs also showed no erythema or oedema [40].

## 3. Discussion

Wound healing is described as a survival mechanism to maintain the normal anatomical structure and function of living tissue after being disrupted by physical, chemical, microbiological, or immunological injury [48]. Our findings showed that the herbal plant *M. oleifera* has positive impacts on the wound healing process when administered orally or topically, which reflects its traditional use as a leaf paste for wound healing in India [22]. Based on the results, the latest study by Ali et al. (2021) [45] showed that the n-hexane extract of *M. oleifera* seeds administered topically to Swiss Albino mice exhibited wound healing activity by achieving complete excision wound closure on the 13th day of treatment compared to control (carbopol hydrogel without *M. oleifera* extract; 70% wound contraction) and standard (5% povidone; 95% wound contraction), which remain unhealed. Similarly, extracts from leaves, which are the most used plant part in the studies, achieved complete or almost complete excision wound closure by day 14 [30,33,35,37]. The findings also showed that topical application of *M. oleifera* aqueous leaf extracts is the most used intervention for wound healing compared to oral or application of other extracts. Topical application is expected to be advantageous due to its local delivery of high and sustained concentrations of active ingredients at the wound site, therefore, contributing towards faster wound contraction, wound closure, and overall healing [49,50]. The bioactive compound from the extract can be released quickly and hasten the transition to the epidermal regeneration process [33]. In addition, local applications are supposed to have lesser systemic absorption than those consumed orally, which may reduce the risk of toxicity. For example, a clinical study comparing the essential oil extracted from leaves of *Melaleuca alternifolia* and benzoyl peroxide showed significantly lower incidence of adverse effects, such as dryness, irritation, itching, and burning, with tea tree oil (44%) than with benzoyl peroxide (79%), although it is shown that *M. alternifolia* can cause allergic contact dermatitis if ingested orally [51].

The right wound area humidity or appropriate moisture is also important to accelerate the formation of the growth factors and increase the fibroblast cell infiltration for wound healing [52]. This can be explained by three mechanisms (keratinocyte proliferation, fibroblast growth, and the preservation of growth factors), which improves wound healing under controlled hydration and a moist environment [53]. In addition to *M. oleifera*, other plant species, such as *Avicennia schaueriana* [54], *Morinda tinctoria* Roxb [55], and *Albizia amara* [56], have also been subjected to wound healing studies, wherein their aqueous leaf extract showed significant wound healing activity.

Most of the included studies reported on and discussed the potential ability of *M. oleifera* in accumulating collagen, the most important protein for wound recovery [9,34,46]. Collagen formation is an important step in wound healing, as synthesized collagen will enhance epithelialization, a key factor in excision wound recovery. For incision wounds, newly synthesized collagen as well as fiber stabilization will increase the tissue tensile strength at the wound site to improve recovery [32,41]. Increased collagen content was also correlated with increased hydroxyproline content, which promotes the healing in dead space wounds [41,43]. Collagen content was calculated by measuring hydroxyproline, which is an amino acid found in collagen fibers of granulation tissue. It is used to estimate the collagen synthesis where high hydroxyproline net weight showed high collagen content to back wound healing [35,43,48]. Minerals and vitamins found in plants were also thought to contribute towards collagen accumulation. Chemical elements, such as copper, that are present in *M. oleifera* leaves have been reported to be directly involved in collagen synthesis, with iron acting as a cofactor. Vitamin C delivers extra strength and stability to tissues by creating bonds between the collagen fibers, while Vitamin A cross-links the collagen and is involved in the proliferation of epithelial cells [35].

The underlying mechanisms for wound healing were probably through antioxidant, anti-inflammatory, and antimicrobial actions by *M. oleifera*, particularly by chemical compounds present in the plant [30,52,57,58]. Foremostly, it can be attributed to its antimicrobial properties to suppress the infection on the wound site that are known to potentially interfere with the healing process [59,60,61]. Many compounds associated with antimicrobial activity were found in this plant, such as glycosides, tannins, triterpenoids, flavonoids, saponins, benzyl isothiocyarate, other isothiocynates, the alkaloid family, secondary metabolites, such as anthraquinones and other phenolic compounds [30,36,45,62,63]. Different extract and plant parts might have higher inhibitory effects on certain microbial species [62,63,64]. The alkaloids family, with nitrogen-containing naturally occurring compounds, showed the ability to intercalate with microbe DNA to suppress microbial infection [30]. For instance, peptide content in *M. oleifera* can cause membrane disruption of several species of *Staphylococcus,* including Methicillin-resistant *Staphylococcus aureus*, as well as *Streptococcus* sp., *Eschericia coli*, and *Enterococcus faecalis* [45]. A quantitative histological evaluation of the animals treated with *M. oleifera* showed that it was capable of stimulating macrophage, which is the most critical cell that induces the progression of the wound healing process. Macrophage is a very active phagocyte that removes foreign bodies, microbes, has a direct effect on granular tissue development, as well as wound regulation, cellular activation via cytokines, and angiogenesis via growth factors [32,65]. The current findings from the included studies show the potential of *M. oleifera* in acute wounds, which consists of open wounds (incisional and excisional model) and closed wounds (dead space). It is recommended to further investigate its potential on non-healing wounds, which is due to a stalled inflammation phase and imbalance of proteases during the tissue formation phase [66]. 

*M. oleifera* leaves, particularly, have been reported to contain phenolics, such as flavonoids and tannins, ascorbic acid, carotenoids, and polyphenolics, such as chlorogenic acid, rutin, quercetin glucoside, and kaempferol rhamnoglucoside, which are a good source of natural antioxidants to protect tissues against oxidation damage [30,67,68]. Natural compounds with polyphenols are known to act as primary antioxidants due to their properties for inactivating lipid free radicals or prevention of the decomposition of hydroperoxides into free radicals by their redox properties [52]. Antioxidant activity is important because it can intervene in the inflammation tissue damage, which is due to the liberation of reactive oxygen species from phagocytes invading the inflammation sites [69,70,71,72]. According to Hosseinkhani et al. (2017) [58], antioxidant properties were found in Persian medicine used for wound healing, which are *Cocos nucifera* L., *Commiphora mukul* (Hook ex Stocks) Engl, *Gentiana lutea* L., *Teucrium polium* L., *Punica granatum* L. *Plantago major* L., *Adiantum capillus-veneris* L., *Aloe vera* (L.) Burm f, and *Potentilla reptans* L.

The ability of *M. oleifera* to induce anti-inflammatory action towards the wound site is due to its ability to antagonize the anti-healing effect of steroids [41]. The action was by stimulating the interleukin-8, an inflammatory α-chemokine, which affects the function and recruitment of various inflammatory cells, fibroblasts, and keratinocytes. Its ability to down-regulate pro-inflammatory cytokines, such as IL-1β, IL-6, and TNF-α, helps accelerate wound healing [73,74]. Quercetin may have been involved in the reduction in the inflammatory process by inhibiting the action of neutral-factor kappa-beta (NF-kβ) and subsequent NF-kβ-dependent downstream events and inflammation [75]. Other anti-inflammatory plant species that showed significant wound healing activities against excision, resutured incision, and dead space wound are *G. lutea* [76], *T. polium* [77], and *C. nucifera* [78]. However, careful consideration of prolonged inflammation is not usually represented in animal models and, therefore, anti-inflammatory action may not be sufficient considering other factors, such as the oxygen, nutrients, bacterial infection, and cellular events [79]. Therefore, it is important to investigate these issues on human wounds measuring the pro-inflammatory and anti-inflammatory cytokine levels together with antioxidant and antimicrobial activities of the plant.

Wound healing studies have also always been associated with antidiabetic activity. *M. oleifera* showed an antidiabetic effect while accelerating wound healing on diabetic-induced animals [32,33,34,47]. The compounds present in *M. oleifera*, such as gallic acid, rutin, and vicenin-2 active compounds, as well as other flavonoids and phenolic metabolites, improved the hyperglycemic condition of diabetic-induced animal models [34,80]. This suggested that coupled with the wound healing effect, *M. oleifera* contains an antidiabetic effect and is, thus, suitable to treat wounds in diabetic patients. It was reported that diabetic patients are more susceptible to wound infection where the infection rate was found 11% higher compared to the general patients’ population [81]. Common infections are usually caused by *E. coli*, *P. aeruginosa*, and *S. aureus*. [30,47].

Despite the results, certain limitations should be addressed. There were three papers excluded as full text was not available and attempts to contact the authors to request the papers were unsuccessful. There is a possibility that certain important data are not fully presented due to the inclusion of English-language articles only. In future, better methodological design in animal studies with a detailed level of reporting is important to improve the risk of bias assessment.

## 4. Materials and Methods

A scoping review of the literature was conducted in accordance with the methodology by Levac et al. [82]. The Preferred Reporting Items for Systematic reviews and Meta-Analyses extension for Scoping Reviews (PRISMA-ScR) guidelines were followed, which are a set of 20 essential items and 2 optional items that were created to help improve the quality, completeness, and transparency of scoping reviews (Table S1) [83].

### 4.1. Identifying the Research Question

This review was conducted based on the primary question “What are the wound healing potentials of *M. oleifera*?”. The secondary questions expanded from this primary question are as follows:

(i). What plant parts of *M. oleifera* are being studied for wound healing efficacy?
(ii). What formulation and route of administration is suitable for *M. oleifera*’s wound healing effect?
(iii). What are the findings of its efficacy on incision, excision, dead space, and other types of wounds?
(iv). What is the safety profile of *M. oleifera* in animal toxicity studies?

### 4.2. Identifying Relevant Studies

A systematic search was conducted by two independent authors for published articles which focus on health and health-related topics, using combination of keywords relating to *M. oleifera* and wound healing. The search strategy used is presented in the Supplementary Material (Table S2). Six electronic databases (i) PubMed; (ii) Google Scholar; (iii) ScienceDirect; (iv) LILACS; (v) ClinicalTrials.gov; (vi) CENTRAL were searched from inception until 30 November 2021. 

### 4.3. Study Selection

All references were imported into EndNote X9, duplicates were removed, and the records were screened for the following criteria: Inclusion criteria: (i) original research that presents *Moringa oleifera* wound healing efficacy; (ii) in vivo/animal models papers; (iii) clinical papers; (iv) full-text articles written in English; (v) no limitations on years of study or publication. Exclusion criteria: (i) in vitro/in silico/modelling papers; (iii) safety/toxicity papers not in the context of a wound healing study. 

### 4.4. Charting the Data

The data-charting process which included screening of title, abstract, and full text was conducted independently by two teams consisting of two pairs of authors and all ambiguities or disagreements regarding the type of data considered for the final selection of publications were discussed together by all five authors.

After screening, extraction was conducted by two pairs of authors who extracted into Excel for the included studies. Since no clinical papers were found, the data extraction is catered to in vivo papers which covers:

- Efficacy: (i) author, year; (ii) plant part used; (iii) type of extraction; (iv) type of animals; (v) route of administration; (vi) dosage and formulation; (vii) comparator; (viii) type of wounds; (ix) findings.
- Safety: (i) author, year; (ii) plant part used; (iii) type of extraction; (iv) type of animals; (v) route of administration; (vi) dosage and formulation; (vii) type of toxicity test; findings.

### 4.5. Data Analysis

Two pairs of authors will independently evaluate the risk of bias in animal studies using the Systematic Review Centre for Laboratory animal Experimentation (SYRCLE) risk of bias tool. These authors will score the risk of bias in each domain and the overall risk will be reported using the Cochrane Review Manager (RevMan, version 5.4) software. (Review Manager 5 (RevMan 5) (Computer Program); Version 5.4; Nordic Cochrane Centre: Copenhagen, Denmark, 2014) The third author will be consulted to resolve any cases of disagreement. The results of these assessments will be presented in a risk of bias summary and assessment figures.

## 5. Conclusions

Based on the in vivo studies, *M. oleifera* wound healing potential with aqueous extracts of *M. oleifera* leaves was found as the most used intervention for wound healing compared to oral or topical application of other extracts. Meanwhile, the n-hexane extract of *M. oleifera* seeds showed the fastest excision-induced wound healing activity. However, the exact phytochemical responsible and the formulation factor, such as particle size and type of extract used, need to be determined to comprehend the complete mechanism of wound healing activity by *M. oleifera* and its role as a therapeutic agent, supplementation, or combination therapy. Further studies also need to be conducted on other wound models and safety assessments to prevent the interferences of other therapeutic actions and unwanted adverse effects in order to yield the best wound healing efficacy.

## Figures and Tables

**Table 1:** Characteristics of included studies.

Author, Year	Country	Plant Part	Types of Formulation/Extract	Animal Model
Akanji, 2015 [30]	Nigeria	Leaves	Methanol	Matured Wistar albino rats
Sivaranjani, 2016 [31]	India	Leaves	Aqueous	Male Wistar albino rats
Azevedo, 2018 [32]	Brazil	Leaves	Aqueous	Diabetic induced adult Wistar rats
Chin, 2018 [33]	Malaysia	Leaves	Aqueous	Diabetic induced Male Sprague Dawley rats
Muhammad, 2016 [34]	Malaysia	Leaves	Aqueous	Diabetic induced male Wistar rats
Kumar, 2016 [35]	India	Leaves	Aqueous	Male Swiss albino rats
Charde, 2011 [36]	India	Leaves	Ethanol	Male Wistar albino rats
Hukkeri, 2006 [37]	India	Leaves	Ethyl acetate and ethanol	Male Wistar rats
Rathi, 2006 [38]	India	Leaves	Aqueous	Male Swiss albino rats
Islam, 2018 [39]	Bangladesh	Leaves	Aqueous	Female Wistar rats
Agnes, 2014 [40]	Philippines	Twig	Ethanol	Healthy Guinea pigs
Lambole, 2012a [41]	India	Bark	Aqueous	Wistar albino rats
Lambole, 2012b [42]	India	Bark	Aqueous and ethanol extract	Wistar albino rats
Rathi, 2004 [43]	India	Fruits (pulp and seed)	Aqueous	Male albino rats
Amaliya, 2019 [44]	Indonesia	Leaves	Ethanol	Male Sprague–Dawley rats
Ali, 2021 [45]	India	Seeds	n-hexane	Swiss albino mice
Ayu, 2020 [46]	Indonesia	Leaves	Not reported	Male Wistar rats
Natarajan, 2018 [47]	Malaysia	Leaves	Aqueous	Diabetic induced male Wistar albino rats

**Table 2:** Data extraction table on wound healing activity.

Author, Year	Mode	Dosage/Formulation	Comparison	Type of Wound	Findings
Akanji, 2015 [30]	Topical	100 mg/mL *M. oleifera* extract	Gentamycin (8 mg/mL)	Excision	Wound closure in the non-infected group was 61.0%, significantly higher compared to gentamycin (21.0%) on day 12th post wound. As for *Staphylococcus aureus* infected group, wound closure was 93.1%, higher than gentamycin (80%). For *Pseudomonas aeruginosa* infected group, complete wound closure achieved comparable to gentamycin. (*p* < 0.05)
					*S. aureus* infected and non-infected group treated with *M. oleifera* showed shorter epithelialization period (16th day and 14th day) compared to *P. aeruginosa* (18th day). (*p* < 0.05)
Sivaranjani, 2016 [31]	Topical	0.5% ointment gel contained TiNPs developed using *M. oleifera*.	Gel contained sulfadiazine	Excision	Wound closure was faster (92.36 ± 0.5%) compared to control (75.23 ± 0.58%) and standard drug (83.55 ± 0.57%) on day 12 post wound. (*p* < 0.05)
Azevedo, 2018 [32]	Oral followed by topical	Oral: 100 mg/kg *M. oleifera* extract	Normal saline	Excision	Wound closure for both normal and diabetic induced rats were faster (92% and 88%, respectively) compared to control (61% and 64%, respectively) (*p* < 0.05)
		Topical: 200 μL of 10% *M. oleifera* extract			
Chin, 2018 [33]	Topical	0.1%, 0.5%, 1.0% (*w*/*v*) *M. oleifera* extract-loaded film	Commercial dressing (Kaltostat)	Excision	Dose 0.5% (*w*/*v*) showed faster wound closure (77.67 ± 7.28%) compared to commercial dressing Kaltostat (28.67 ± 12.83%) on day 7 post wound. (*p* < 0.05)
				Abrasion	Wound closure was faster (81 ± 4.5%) compared to control (28.8 ± 9.85%) and commercial dressing (73.13 ± 8.05%) on day 3 post wound (*p* < 0.05).
Muhammad, 2016 [34]	Topical	0.5, 1.0, and 2.0% (*w*/*w*) *M. oleifera* ointment	Silver sulfadiazine	Excision	The highest dose (2%) showed faster contraction rate from 59.7% on day 3 progressed to complete wound closure by day 21 compared to normal and diabetic control. The epithelialization period recorded in the aqueous fraction treated group was 11 ± 1 days compared to untreated diabetic control (15 ± 1 days).
Kumar, 2016 [35]	Topical	*M. oleifera* extract paste	Untreated wound	Excision	Wound closure was achieved by 14th day of treatment (99.3 ± 0.09%) compared to control (88.0 ± 0.54%) and the mean period of epithelization shorter (14.66 days) compared to control (17.16 days) (*p* < 0.001).
				Incision	Tensile strength was higher (507.5 ± 7.14 g) compared to control (367.5 ± 6.76 g) (*p* < 0.001)
Charde, 2011 [36]	Topical	*M. oleifera* extract	Framycetine Sulphate Cream (FSC)	Excision	Wound closure was (98.52%) on day 27 post-wound compared to control (78.61%) and FSC (100%). The epithelialization showed higher similarity to normal tissues (4.06 ± 0.04) compared to control (1.23 ± 0.55) and comparable to FSC (4.16 ± 0.04) where value 5 refers to maximum similarity and 0 refers to least similarity. (*p* < 0.001)
				Incision	Tensile strength of extract and FSC were almost comparable with 320.51 ± 0.45 g and 365.41 ± 3.55 g, respectively. (*p* < 0.001)
				Dead space	Granuloma breaking strength for the extract was higher than control (315.67 ± 1.55 g, 278.89 ± 2.60 g, respectively) (*p* < 0.001)
Hukkeri, 2006 [37]	Topical ointment and oral	Topical: 10% (*w*/*w*) *M. oleifera* ointment Oral: 300 mg/kg *M. oleifera* extract	Vicco turmeric cream	Excision	Ethyl acetate extract significantly showed faster wound closure (99.87 ± 0.42%) than ethanol extract (99.69 ± 0.45%) comparable to Vicco turmeric cream (99.90 ± 0.32%) on day 14 post wound. (*p* < 0.001)
	Oral	300 mg/kg *M. oleifera* extract	Vicco turmeric cream	Incision	Ethyl acetate extract significantly showed higher tensile strength (473.80 ± 1.23) than control (241 ± 1.02) and ethanol extract (439.17 ± 1.11) respectively. (*p* < 0.001)
				Dead space	Granuloma studies showed the tensile strength of ethyl acetate extract was significantly higher (355.83 ± 0.89 g) compared to control (180.00 ± 0.98 g) and ethanol extract (345.00 ± 0.86 g) respectively. (*p* < 0.001)
Rathi, 2006 [38]	Oral	300 mg/kg bw *M. oleifera* extract	2% gum acacia	Excision	Almost complete wound closure achieved on day 16 post wound (99.92 ± 0.70%), faster than 2% gum acacia (83.52 ± 1.78%). Epithelialization period was significantly shorter compared to 2% gum acacia. (*p* < 0.05)
				Incision	Tensile strength was significantly higher (358.50 g ± 8.03) compared to 2% gum acacia (282.66 g ± 0.24). (*p* < 0.05)
				Dead space	Tensile strength, hydroxyproline content, and granuloma weight were significantly higher (252.0 ± 6.54 g, 6.83 ± 0.13 µg/300 mg, and 45.61 ± 1.85 mg%, respectively) than 2% gum acacia (219.0 ± 5.70 g, 5.23 ± 0.20 µg/300 mg and 36.72 ± 1.90 mg%, respectively). (*p* < 0.05)
Islam, 2018 [39]	Topical	2% extract *M. oleifera* gel (MO), amniotic membrane gel (AM), and AM + MO	Untreated wound	Burn induced	Wound closure for AM+MO was the fastest (96 ± 1.96%) compared to control (43.45 ± 1.32%) on day 24 and showed shorter epithelialization period (19.6 days) compared to AM (23.2 days), MO (28.2 days) and control (31.4 days). (*p* < 0.05)
Agnes, 2014 [40]	Topical	5, 7.5, 10 mg/mL *M. oleifera* extract patch	Calmoseptine	Excision	Dose 10 mg/mL stimulated the wound healing property of the standard drug used. (*p* < 0.05)
Lambole, 2012a [41]	Topical	5% *w*/*w M. oleifera* ointment	5% *w*/*w* povidone iodine ointment (PIO)	Excision	Contrary with the DMS treated group, the extract showed complete wound closure on day 20 compared to control simple ointment (94.00 ± 0.44%), standard drug PIO (97.17 ± 0.5%), DMS injection (86.00 ± 0.57%) and extract treatment after DMS injection (95.50 ± 0.71%). The epithelialization period was shorter (13.83 ± 0.47 days) than control (21.17 ± 0.30 days) and standard (17.83 ± 0.47 days) respectively. (*p* < 0.001)
				Incision	The wound breaking strength was significantly higher (556.30 ± 1.28 g) compared to control (388 ± 0.98 g), PIO (492 ± 2.37 g), and that prior injection with DMS. (*p* < 0.001)
				Dead space	The extract significantly increased the granuloma breaking strength (521.70 ± 2.47 g) and hydroxyproline content (65.03 ± 0.80 µg/mL) compared to control (262.20 ± 4.00 g and 33.63 ± 1.17 µg/mL, respectively). (*p* < 0.001)
Lambole, 2012b [42]	Topical	5% *w*/*w M. oleifera* ointment	5% (*w*/*w*) Povidone iodine ointment	Excision	Aqueous extract showed the fastest wound contraction progress by 12th day with 90.17 ± 0.54% while ethanol extract was 88.17 ± 0.47% compared to PIO (65.17 ± 0.83%) and control (65.17 ± 0.83%). (*p* < 0.001) Epithelialization time of aqueous and ethanol extract decreased significantly to 13.83 and 15.83 days, respectively compared to control (21.17 days). (*p* < 0.001)
				Incision	Wound breaking strengths of aqueous extract was the best (556.3 ± 1.28 g) compared to ethanol extract (519.7 ± 1.28 g), PIO (492.8 ± 2.37 g) and control (388.3 ± 0.98 g). (*p* < 0.001)
				Dead space	Granuloma breaking strength of aqueous extract was higher (521.7 ± 2.47 g) compared to PIO (412.0 ± 5.85 g) and others. (*p* < 0.001) Hydroxyproline content of aqueous extract was higher (65.03 ± 0.80 µg/mL) compared to PIO (48.60 ± 0.41) and others. (*p* < 0.001)
Rathi, 2004 [43]	Oral	300 mg/kg *M. oleifera* extract	2% gum acacia	Excision	Extract significantly enhanced wound closure (15.0 ± 0.56 days), reduced mean scar area (27.66 ± 1.87 mm^2^) compared to 2% gum acacia as a control (18.5 ± 0.22 days and 42.33 ± 2.40 mm^2^, respectively). (*p* < 0.001)
				Incision	Wound breaking strength was higher (360.50 ± 8.03 g) compared to control (279.66 ± 0.24 g). (*p* < 0.05)
				Dead space	Granuloma breaking strength (263.0 ± 6.54 g), hydroxyproline content (7.63 ± 0.13 µg/mg), and granuloma weight (0.140 ± 0.0214 g/100 bw) was higher compared to control (209.0 ± 5.70 g, 5.23 ± 0.20 µg/mg), and 0.017 ± 0.0035 g/100 bw, respectively). (*p* < 0.05)
Amaliya, 2019 [44]	Topical	2% & 4% *M. oleifera* gel	Povidone iodine gel 10%	Excision (Palatal wound)	Enhanced wound closure and epithelialization that were shown through increased fibroblast synthesis and increased collagen deposition compared to control.
Ali, 2021 [45]	Topical	5% and 10% of *M. oleifera* hydrogel	Control placebo carbopol hydrogel & standard 5% Povidone	Excision	Wounds healed up to 97% and 98% on day 12 using 5% and 10% hexane hydrogel as compared to standard which is healed by 82%.
				Incision	Tensile breaking strength for both 5% hexane hydrogel and 10% hexane hydrogel (152 g and 156 g, respectively) were significantly higher compared to control (96 g) and standard (115 g). (*p* < 0.01).
Ayu, 2020 [46]	Topical	Not mentioned	Hydrogel	Incision	Epithelialization is significantly enhanced (57.94 ± 7.67 µm) compared to the control group (25.19 ± 3.31 µm) on day 11 post wound. (*p* < 0.01)
Natarajan, 2018 [47]	Topical	0.5%, 1.0%, 2.0% *M. oleifera* hydrogel	Unclear as only ‘market sample’ is mentioned.	Excision	Wound closure was significantly higher (89.76%) compared to control (45.75%) and market sample (73.38%) on day 8 of treatment. (*p* < 0.05)

## Data Availability

Not applicable.

## Supplementary Materials

The following supporting information can be downloaded at: https://www.mdpi.com/article/10.3390/molecules27175541/s1, Table S1: PRISM Checklist; Table S2: Search Strategies Used; Table S3: Qualitative, quantitative and standardization details of herbal interventions.
