# Supplementary material for: Scoping Review: Evaluation of Moringa oleifera (Lam.) for Potential Wound Healing in In Vivo Studies
Source: Molecules. 2022 Aug 28;27(17):5541. doi: 10.3390/molecules27175541 (PMC9457785; doi:10.3390/molecules27175541)
Supplement: Supplementary file 1 [file molecules-27-05541-s001.zip › Table S2 Search Strategies Used.pdf]

S2: Search strategies used

No	Databases	Years of search result (by default mode)	Keywords used
1	PubMed	2006 – 2022	(*Moringa oleifera* OR Merunggai OR drumstick tree) AND (wound OR injury OR lesion OR laceration OR cut OR surgery)
2	Google Scholar	2004 – 2022	
3	ScienceDirect	2013 – 2022	
4	LILACS	2022	
5	ClinicalTrials.gov	No hit	
6	CENTRAL	No hit	
