# Supplementary material for: Scoping Review: Evaluation of Moringa oleifera (Lam.) for Potential Wound Healing in In Vivo Studies
Source: Molecules. 2022 Aug 28;27(17):5541. doi: 10.3390/molecules27175541 (PMC9457785; doi:10.3390/molecules27175541)
Supplement: Supplementary file 1 [file molecules-27-05541-s001.zip › Table S3_Qualitative, quantitative and standardization details of herbal interventions.pdf]

S3: Qualitative, quantitative and standardization details of herbal interventions

No	Author / Year	Intervention formulation	Voucher specimen deposition	Qualitative analysis of content	Quantitative analysis of content	Standardization of intervention
1	Akanji, 2015	Methanolic extract of *M. oleifera* leaves	FHI 108885, Forest Herbarium, Ibadan (FHI)	No	No	No
2	Sivaranjani, 2016	0.5% of titanium nanoparticles gel-based ointment containing aqueous extract of *M. oleifera* leaves	No	No	No	No
3	Azevedo, 2018	Aqueous extract of *M. oleifera* leaves	No	No	No	No
4	Chin, 2018	Aqueous extract-loaded films of *M. oleifera* leaves	HF133, Herbarium UKM Bangi campus, Malaysia	No	No	No but it is mentioned standardised in previous study
5	Muhammad, 2016	Aqueous fraction of matured *M. oleifera* leaves	SK 1561/10, Herbarium Institute of Bioscience, Malaysia	No	No	No
6	Kumar, 2016	Aqueous extract of *M. oleifera* leaves	No	No	No	No
7	Charde, 2011	Ethanolic extract of *M. oleifera* leaves	No reference number but authenticated in Department of Botany Rashtrasant Tukdoji Maharaj Nagpur University Campus, Nagpur	No	No	No
8	Hukkeri, 2006	Ethanolic extract and ethyl acetate of *M. oleifera* leaves	No reference number but authenticated in the Department of Botany, Karnataka	No	No	No

			University, Dharwad			
9	Rathi, 2006	Aqueous extract of *M. oleifera* leaves	No reference number but authenticated in Botany Department, Agharkar Research Institute, Pune	No	No	No
10	Islam, 2018	Matured *M. oleifera* leaves derived gel with human amniotic membrane, sodium carboxymethylcellulose, propylparaben, glycerin, distilled water, and triethanolamine	No	No	No	Not mentioned but every 2% of *M. oleifera gel* contain 2g of *M. oleifera*
11	Agnes, 2014	Ethanolic extract of *M. oleifera* twigs	No reference number but authenticated at The National Museum, Botany Department, Philippines	No	No	No
12	Lambole, 2012a	Aqueous extract of *M. oleifera* bark	No reference number but authenticated at Hemchandra North Gujarat University, Patan, India	No	No	No
13	Lambole, 2012b	Aqueous and ethanol extract of *M. oleifera* bark	No reference number but authenticated at Hemchandra North Gujarat University, Patan, India	No	No	No
14	Rathi, 2004	Aqueous extract of *M. oleifera* dried pulp and seeds	No reference number but authenticated at Department of Botany, Raja Lakhamagouda Institute of Science, Belgaum, India	No	No	No

15	Amaliya, 2019	Topical gel of ethanolic extract of *M. oleifera* leaves	049/HB/02/2017, Herbarium of Plant Taxonomy Laboratory, Department of Biology, Faculty of Math and Nature Sciences, Universitas Padjadjaran Jatinangor, West Java, Indonesia	No	No	No
16	Ali, 2021	n-hexane extract and hydrogels of *M. oleifera* seeds	No	No	No	No
17	Ayu, 2020	Extract gel of *M. oleifera* leaves containing contained 2.5g CMC Na, 10g Propilen glikol, 1.2g Metylparaben, and additional aquadest until reach 100 gram in weight	No	No	No	No
18	Natarajan, 2018	Hydrogel of *M. oleifera* matured leaves	SK 1561/10, Herbarium of Institute of Bioscience, Malaysia	No	No	No
